# Palmitoylethanolamide (PEA) for Prevention of Gastroesophageal Inflammation: Insights from In Vitro Models

**DOI:** 10.3390/life14101221

**Published:** 2024-09-24

**Authors:** Renato Spigarelli, Carlo Calabrese, Enzo Spisni, Sara Vinciguerra, Ilaria Maria Saracino, Nikolas Kostantine Dussias, Eleonora Filippone, Maria Chiara Valerii

**Affiliations:** 1 Department of Biological, Geological and Environmental Sciences, University of Bologna, Via Selmi 3, 40126 Bologna, Italy; renato.spigarelli@studio.unibo.it (R.S.); enzo.spisni@unibo.it (E.S.); sara.vinciguerra@studio.unibo.it (S.V.); 2 IBD Unit, IRCCS, Azienda Ospedaliero-Universitaria di Bologna, University of Bologna, Via Massarenti 9, 40138 Bologna, Italy; carlo.calabrese2@unibo.it (C.C.); nikolas.dussias@studio.unibo.it (N.K.D.); eleonora.filippone2@unibo.it (E.F.); 3 Department of Medical and Surgical and Sciences, University of Bologna, Via Massarenti 9, 40138 Bologna, Italy; 4 Microbiology Unit, IRCCS, Azienda Ospedaliero-Universitaria di Bologna, University of Bologna, Via Massarenti 9, 40138 Bologna, Italy; ilariamaria.saracino@studio.unibo.it

**Keywords:** gastroesophageal reflux disease (GERD), palmitoylethanolamide (PEA), *Zingiber officinale*, *Musa × paradisiaca*, *Opuntia ficus-indica*, *Olea europaea*

## Abstract

Gastroesophageal reflux disease (GERD) is a digestive disorder that can lead to chronic mucosal damage, causing esophagitis, Barrett’s esophagus and esophageal cancer. GERD currently affects about 13% of the world’s population and represent a major public health concern due to the increasing prevalence and incidence. The aim of this study was to explore complementary strategies for GERD management based the natural compound palmitoylethanolamide (PEA), alone or associated with plant extracts with demonstrated anti-GERD activity (*Zingiber officinale*, *Musa × paradisiaca*, *Opuntia ficus-indica* and *Olea europaea*). For this purpose, two in vitro models based on the esophageal mucosa CP-B cell line were chosen. The first one was based on the exposure of esophageal cells to HCl, while the second one was based on lipopolysaccharide (LPS) treatment to cause a strong inflammatory cell response. Inflammation induced was assessed using a Luminex^®^ assay, measuring the secretion of IL-1β, IL-6, IL-10, IL-8 and TNF-α. Results obtained demonstrate that PEA strongly decreased the inflammatory response elicited by HCl exposure. Moreover, the effect of PEA was enhanced by the presence of natural extracts of *Zingiber officinale*, *Musa × paradisiaca*, *Opuntia ficus-indica* and *Olea europaea*. PEA should be considered as an anti-GERD natural compound of interest.

## 1. Introduction

Gastroesophageal reflux disease (GERD) is a disorder of the gastrointestinal tract caused by the movement of the gastric juice from the stomach to the esophagus which causes mucosal damage and symptoms such as heartburn, chest pain, nausea and chronic cough [1]. This condition can lead to chronic mucosal inflammation (esophagitis), to a metaplastic mucosal lesion, namely Barrett’s esophagus, or to esophageal cancer [2]. Several factors can be involved in GERD onset; in addition to hiatal hernia and inappropriate relaxations of the lower esophageal sphincter (LES), an increased intra-abdominal pressure (obesity), a slowed emptying of the stomach, gastric hyperacidity and a compromised mucosal barrier can contribute to the development of this disorder [2,3]. Diet and lifestyle also play key roles in the manifestation of GERD symptoms. An unbalanced diet with a large intake of acidic beverages, alcohol, coffee, chocolate and spicy and fatty foods can promote the onset of GERD and worsen its symptoms. Abundant meals, especially before bedtime, can increase the disease’s occurrence [4,5].

Basing on a study carried out in 2018, about the 13% of the world’s population shows symptoms characteristic of GERD, with large differences among the different countries examined [6]. GERD incidence and prevalence is increasing all over the world [7], with peaks in disease prevalence (>25%) in South Asian and Eastern European countries, and a lower rate (<10%) in Southeast Asian Countries. In Western Europe and the United States, the incidence rates range between 15% and 20% [6].

Due to its chronic nature and its high prevalence, GERD has a significant economic burden on patients, health services and society and can have a high impact on the quality of life of affected patients [1].

Regardless of the symptoms, we can distinguish two subtypes of GERD that can be discriminated by endoscopic and histological examinations: erosive disease (ERD) and non-erosive disease (NERD). ERD is characterized by lesions of the esophageal mucosa that, in some cases, can evolve in Barrett’s esophagus metaplasia and esophageal cancer. Overall, the prevalent form is NERD, which accounts for about 80% of all GERD [8]. It is still unclear why some patients develop esophageal mucosal erosions while others show no signs of mucosal damage, but this difference could reside in the esophageal mucosal defense mechanisms rather than in the frequency and severity of the acid insult [9]. 

The most widely used drugs for GERD management are proton pump inhibitors (PPIs) and histamine-2 receptor antagonists (H2RAs), which are able to induce symptoms to relieve and reduce esophageal damage by decreasing the production of hydrochloric acid, thereby increasing pH in the stomach. In adults, both therapies have been proven to be effective in GERD. Compared with PPIs, H2RAs are slightly less effective in increasing gastric pH, in healing esophagitis and in preventing ulcers from bleeding [10,11].

Despite the high efficacy, several side effects can be associated with the chronic use of these therapies, such as mineral malabsorption, B12 vitamin deficiency, intestinal dysbiosis and an increased susceptibility to gastroenteritis [12]. Moreover, a higher risk of pneumonia, major cardiovascular events, dementia, bone fractures, gastric cancer and kidney injury has been observed in long-term observational studies [13]. Although the real risks associated with the chronic use of these drugs are still a matter of debate [13], given the uncertainty about long-term safety, these therapies are recommended for periods no longer than 4–8 weeks except in case of severe disease such as erosive esophagitis or Barrett’s esophagus [14]. These limitations make it difficult to therapeutically manage or prevent disease flare-ups, and for this reason, several studies are focusing on the effectiveness of natural compounds for the management of reflux disease. Plant extracts have been historically used in traditional medicine for the management of gastrointestinal disorders. Among these, ginger (*Zingiber officinale*) stands out due to its demonstrated anti-inflammatory, antiacid and prokinetic activities that have been attributed to the presence of alginates and gingerols [15]. Other plants extract such as banana (*Musa × paradisiaca*) showed protective effects on the gastric mucosa [16,17] as did extracts of prickly pear (*Opuntia ficus-indica*) cladodes and olive (*Olea europaea*) leaves. The latter were recently tested in a randomized double-blinded placebo controlled clinical trial and showed a fair amount of efficacy in the treatment of GERD [18,19].

Recently, palmitoylethanolamide (PEA), isolated from soy lecithin, peanuts and egg yolk, has raised great interest due to its beneficial effects in gastrointestinal disorders such as irritable bowel syndrome (IBS) and inflammatory bowel diseases (IBDs) [20]. For this reason, PEA could be considered a natural compound of interest for the long-term management of GERD, but specific studies on the effects of PEA in GERD are lacking.

In this study, we analyzed the anti-inflammatory effects of PEA alone or in association with a mix of *Opuntia ficus-indica* cladode and *Olea europaea* leaf extract, namely Mucosave^®^ (OO) (Bionap SRL, Catania, Italy), with *Musa × paradisiaca* fruit extract (MP) and with *Zingiber officinale* rhizome extract (ZN). This study was conducted with two different in vitro models of GERD based on the CP-B esophageal mucosa cell line.

The aim of the study was to evaluate the anti-inflammatory effects of PEA in vitro on an esophageal cell line (CP-B). PEA was tested alone and in combination with other phytotherapeutics already known for their positive effects on GERD, assessing the possibility of a future use of PEA in the treatment of the disease.

## 2. Materials and Methods

### 2.1. Study Treatments and Dose Selection

**Figure 1:**
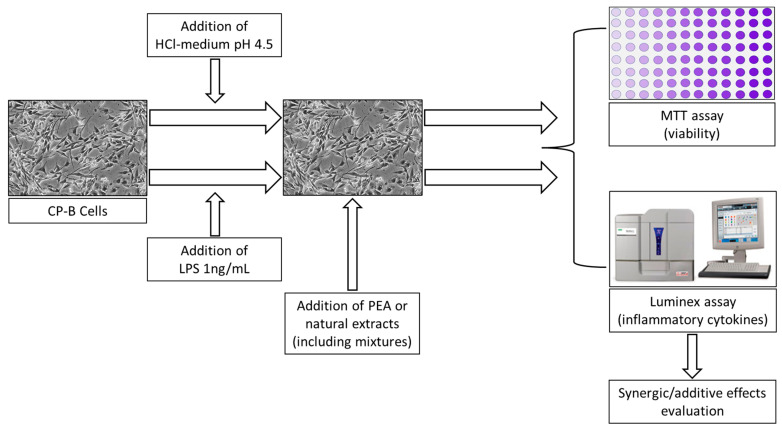
Scheme of the experimental design.

The experimental design is shown in Figure 1. All the experiments were conducted on the CP-B cell line, treated with HCl or lipopolysaccharide (LPS) to induce an inflammatory response. PEA and the other natural compounds were then added in the concentrations and combinations described in Table 1. After treatments, a cell count, viability assay and cytokine analysis on cell-conditioned media were performed to evaluate the anti-inflammatory activity of PEA alone and in combination with OO, MP and ZN.

PEA (CAS No.: 544-31-0) was purchased from Merck KGaA (Darmstadt, Germany) and used at concentration of 3 μg/mL and 15 μg/mL [21].

Mucosave^®^ is a solid blend of extracts from *Opuntia ficus-indica* cladodes (32–35% *w*/*w*) and *Olea europaea* leaf extract (23–25% *w*/*w*). This extract has been characterized by the producer and contains vitamins (C, E and B group), minerals (including calcium, magnesium and potassium), polysaccharides (soluble fiber), phenols, flavonoids and betalains. It was purchased from Bionap SRL (Catania, Italy) and used at concentrations of 1 mg/mL and 3 mg/mL.

*Musa × paradisiaca* fruit extract, obtained by a spray-drying process, contains vitamins (C, A and B group), minerals (including potassium, magnesium, calcium and iron), carbohydrates, dietary fiber, phenolic compounds and flavonoids, phytosterols, amino acids (with tryptophan being the main one); and organic acids such as malic acid and citric acid. It was purchased from NaturMed Scientific GmbH (Frankfurt, Germany) and used at concentrations of 1 mg/mL and 3 mg/mL.

*Zyngiber officinalis* rhizome extract (6-gingerol; >10 g/Kg) was purchased from Greenfield Botanicals SRL (Asti, Italy). Its main components are gingerols, shogaols, essential oil (containing zingiberene, bisabolene, curcumene and farnesene), polyphenols, vitamins (C and B group), minerals (including potassium, magnesium and manganese), dietary fiber and essential amino acids. It was used at concentrations of 250 μg/mL and 500 μg/mL [22].

The concentrations of PEA, OO, MP and ZN were set using the minimum and maximum concentrations that have been shown to be effective in previous studies published in the literature. To evaluate possible synergistic effects between the compounds, we performed an analysis of the anti-inflammatory effects of binary, ternary and quaternary mixtures of the compounds as described in Table 1. For binary, ternary and quaternary mixtures, the minimum concentrations that have been shown to be effective in reducing inflammation in our model were used.

### 2.2. Cell Cultures

CP-B esophageal cells derived from human Barrett’s esophageal metaplasia (ATCC; Manassas, VA, USA), were grown in bronchial epithelial cell medium with 20 ng/mL of epidermal growth factor (EGF), 20 mg/mL of adenine, 140 μg/mL of bovine pituitary extract and L-glutamine 4 mM (BEGM BulleKit, Lonza Group AG, Basel, Switzerland) enriched with 10% fetal bovine serum (FBS, Merck KGaA, Darmstadt, Germany). Morphology of cultured CP-B cells is shown in Supplementary Figure S1. Cells were grown according to ATCC standard protocols and finally seeded in 24-well, flat-bottom plates before the experiments. Cells had grown to form a monolayer inside the wells before adding treatments. Inflammation was induced by exposing cells to a HCl-acidified growth medium buffered with HEPES (HCl 100 mM, pH 4.5) [23] or by exposing cells to the bacterial toxin LPS from *E. coli* (strain 0111: B4, CliniSciences LTD, Nanterre, France) at a concentration of 1 ng/mL.

To test the efficacy of our treatments in the modulation of HCl-induced inflammation, cells were cultured in HCl-acidified medium for 15 min. After treatment, cells were rinsed twice with standard medium (pH = 7.2) and incubated in fresh standard medium alone or supplemented with the treatments under investigation as described above (Table 1) for 12 h.

To test the efficacy of our treatments in the modulation of LPS-induced inflammation, cells were cultured in standard medium containing LPS alone or in combination with the treatments under investigation, as described above, for 12 h.

After treatment, conditioned media were collected, centrifuged at 16,000× *g* and stored at −80 °C for subsequent cytokine analysis.

In all experiments, CP-B cell number and viability was assessed after treatment by using the Luna-FL cell counter (Logos Biosystem, Gyeonggi, South Korea) and the MTT [3-(4,5-dimethylthiazol-2-yl)-2,5-diphenyl-2H-tetrazolium bromide] assay, respectively.

### 2.3. MTT Vitality Assays

After treatments, cell viability was detected using the MTT assay, according to the ISO 10993-5 International Standard procedure (ISO 10993-5, 2009) as previously described [24]. The MTT substrate was prepared in bronchial epithelial cell medium, added to cells in culture to attain a final concentration of 1 mg/mL and then incubated for 2 h at 37 °C with 5% CO_2_. After incubation, the medium was removed by aspiration. Isopropanol (100 µL) was added to each well, and formazan dye formation was evaluated by a multi-well scanning spectrophotometer at 540 nm. Data are expressed as a percentage of viability when compared with untreated cells (negative control—considered as 100% viability). Positive controls were obtained by treating cells with 1% Triton X-100 (considered as 100% cell death). For background controls, blank wells with growth medium without cells were used.

### 2.4. Cytokine Analysis

Cytokines secreted by CP-B cells (IL-1β, IL-6, IL-10, IL-8 and TNF-α) were measured by using a multiplex Luminex^®^ reader (BioPlex 200) (Bio-Rad Laboratories Inc., Hercules, CA, USA) and a customized human magnetic Luminex^®^ screening assay (R&D Systems, Bio-Techne Corporation, Minneapolis, MN, USA), according to the manufacturer’s protocol. The concentration of cytokines was based on the same number of cells (in monolayer they reach 250,000 per well) and on the evaluation of cell number and viability, as measured with the MTT assay. All experimental conditions were analyzed in quadruplicate.

### 2.5. Synergy Analysis

Synergy analysis and the calculation of a combination index (Ci) was performed with the validated Web application SynergyFinder (Network Pharmacology for Precision Medicine in the Research Program of System Oncology, Faculty of Medicine at the University of Helsinki, Helsinki, Finland).

The combination index (Ci) is closely related to the Loewe model, a mathematical model used to interpret drug combinations, implemented in SynergyFinder [25]. The results offer quantitative definition for additive effects (Ci = 1), synergism (Ci < 1), and antagonism (Ci > 1) in drug combinations.

### 2.6. Statistical Analysis

Statistical analysis was performed using the two-tailed unpaired t-test after F-test analysis for verifying the normal distribution, by using GraphPad 9 software (San Diego, CA, USA). Data are expressed as means ± standard deviation (S.D.). Experiments were performed in quadruplicate (n = 4). Differences between samples were considered statistically significant at *p* < 0.05.

## 3. Results

### 3.1. Effect of Study Treatments on Cell Viability

No significant effects on cell viability were detected after treating CP-B cells with PEA and natural extracts of OO, MP and ZN at the selected doses. CP-B cell number and viability were not affected after treatment with HCl or LPS.

### 3.2. Effect of LPS and HCl Exposure on Inflammatory Cytokine Secretion

HCl exposure resulted in a significant 2-fold increase in IL-6 secretion, a 2.5-fold increase in IL-1β secretion and a 2-fold increase in IL-8 secretion. No effects were detectable on TNF-α and IL-10 secretion, which remained below the detection sensitivity of the instrument.

LPS treatment resulted in a 32-fold increase in IL-6 secretion, a 28-fold increase in IL-1b secretion, a 25-fold increase in IL-8 secretion, a 7-fold increase in TNF-α and a 7-fold increase in IL-10 secretion.

### 3.3. Effect of PEA, OO, MP and ZN on Cytokine Secretion in the HCl-Induced GERD Model

**Figure 2:**
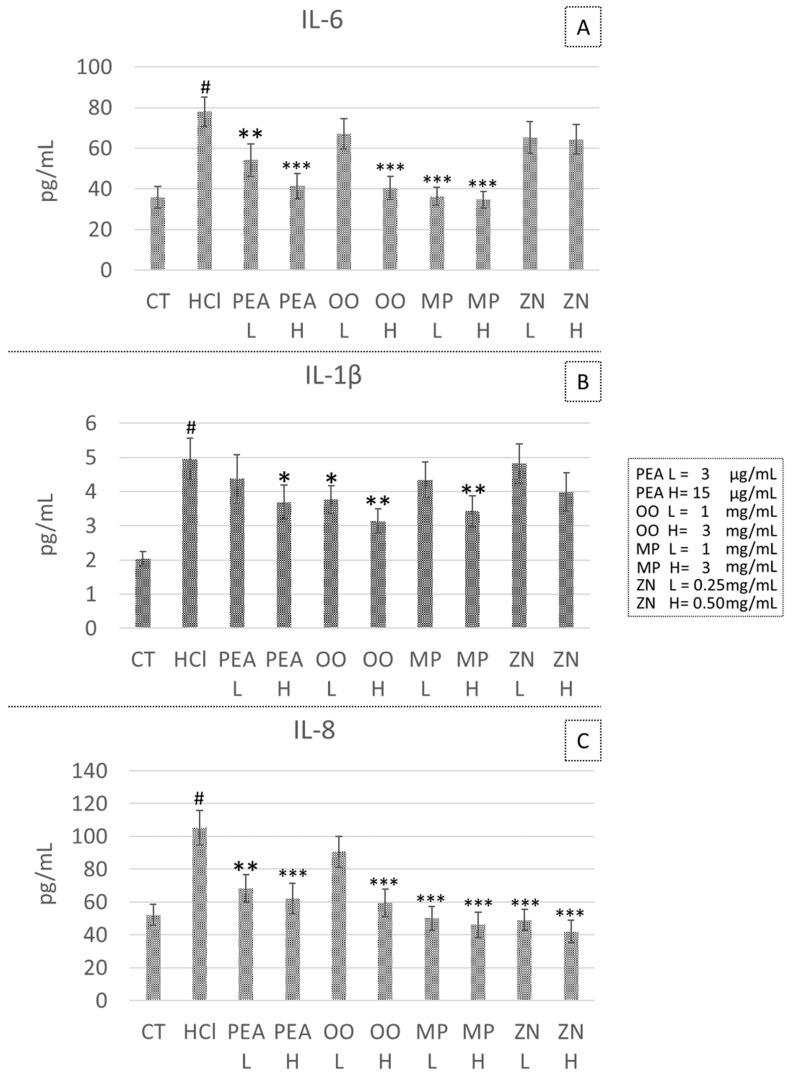
Effects of the tested compounds on the concentration of the pro-inflammatory cytokines IL-6 (**A**), IL-1β (**B**) and IL-8 (**C**) after acid exposure. CT, control; HCl, acid-exposed positive control; PEA, palmitoylethanolamide, OO, *Olea europaea* and *Opuntia ficus-indica* extract; MP, *Musa × paradisiaca*; ZN, *Zingiber officinale*. H, high and L, low concentration values, indicated in the figure legend. * *p* < 0.05, ** *p* < 0.01 and *** *p* < 0.001 compared to the HCl stimulated positive control. # *p* < 0.001 compared to the negative untreated control (CT).

PEA treatment significantly reduced IL-6 secretion at both tested doses (−30.6% at 3 μg/L, *p* = 0.019 and −46.9% at 15 μg/L, *p* = 0.003), IL-1β secretion was significantly reduced only at the higher dose tested (−25.6% at 15 μg/L, *p* = 0.048) and IL-8 secretion was reduced at both doses tested (−35.3% at 3 μg/L, *p* = 0.009; −41.1% at 15 μg/L, *p* = 0.006). OO treatment significantly decreased IL-6 secretion at the higher dose tested (−48.1% at 3 mg/mL, *p* = 0.002), a significant decrease of IL-1β at both the doses tested (−24.2% at 1 mg/mL, *p* = 0.045 and −36,7% at 3 mg/mL, *p* = 0.010) and a significant decrease of IL-8, only at the higher dose tested (−43.7% at 3 mg/mL, *p* = 0.004). MP treatment significantly decreased IL-6 secretion at both the doses tested (−53.4% at 1 mg/mL, *p* = 0.002 and −55.5% at 3 mg/mL, *p* = 0.001), significantly decreased IL-1β secretion at the higher dose tested (−30.8% at 3 mg/mL, *p* = 0.024), and significantly decreased IL-8 secretion at both doses tested (−52.5% at 1 mg/mL, *p* = 0.002; −56.3% at 3 mg/mL, *p* = 0.001). No significant effect on cytokine secretion was observed after ZN treatment at both doses tested (Figure 2).

### 3.4. Effect PEA, OO, MP and ZN on Cytokine Secretion in the LPS-Induced Inflammatory Model

**Figure 3:**
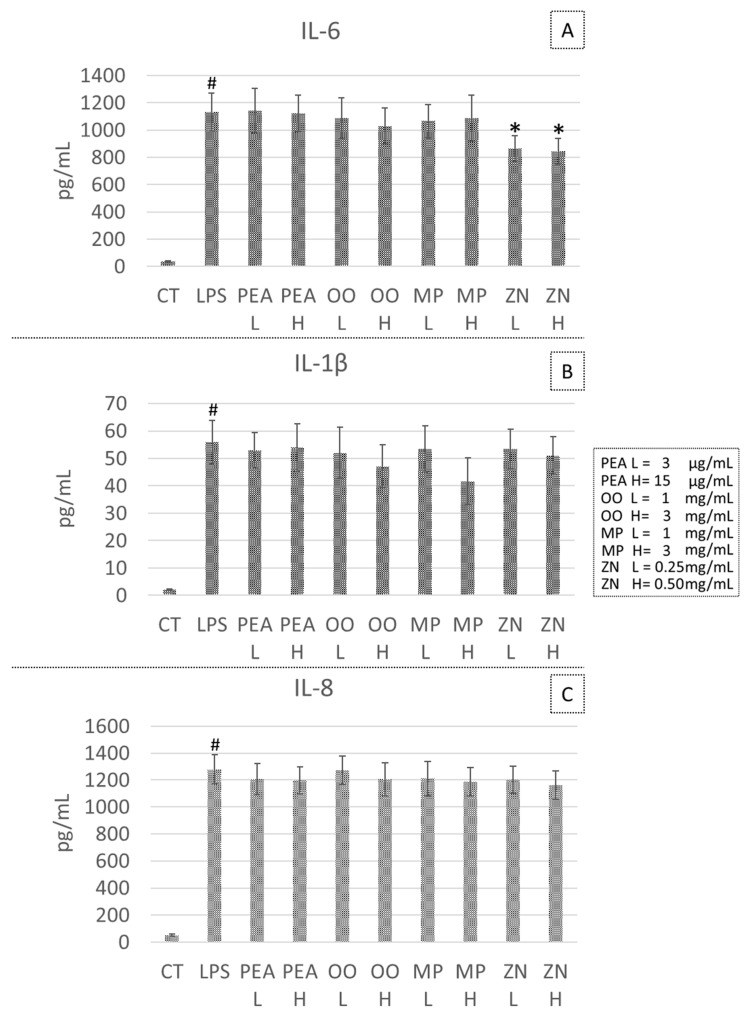

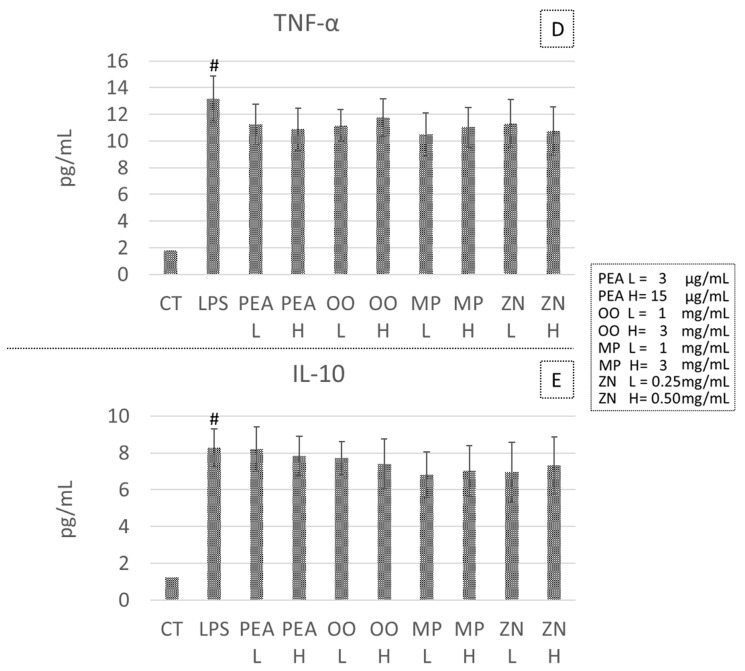
Effects of the tested compounds on the concentration of the pro-inflammatory cytokines IL-6 (**A**), IL-1β (**B**), IL-8 (**C**), TNF-α (**D**) and IL-10 (**E**) after LPS stimulation. CT, control; LPS, Lipopolysaccharide positive control; PEA, palmitoylethanolamide, OO, *Olea europaea* and *Opuntia ficus indica* extract; MP, *Musa × paradisiaca*; ZN, *Zingiber officinale*. H, high and L, low concentration values are indicated in the figure legend. * *p* < 0.05 compared to the HCl stimulated positive control. # *p* < 0.001 compared to the negative untreated control (CT).

None of the treatments used was able to modulate cytokine secretion in the LPS-induced inflammation except for a significant decrease in IL-6 secretion, observed after ZN treatment at both tested doses (−23.7% at 0.25 mg/mL, *p* = 0.048; and −25.5% at 0.50 mg/mL, *p* = 0.041) (Figure 3).

### 3.5. Effects of Combined PEA, OO, MP and ZN on Cytokine Secretion in HCl- and LPS-Induced Inflammation

**Figure 4:**
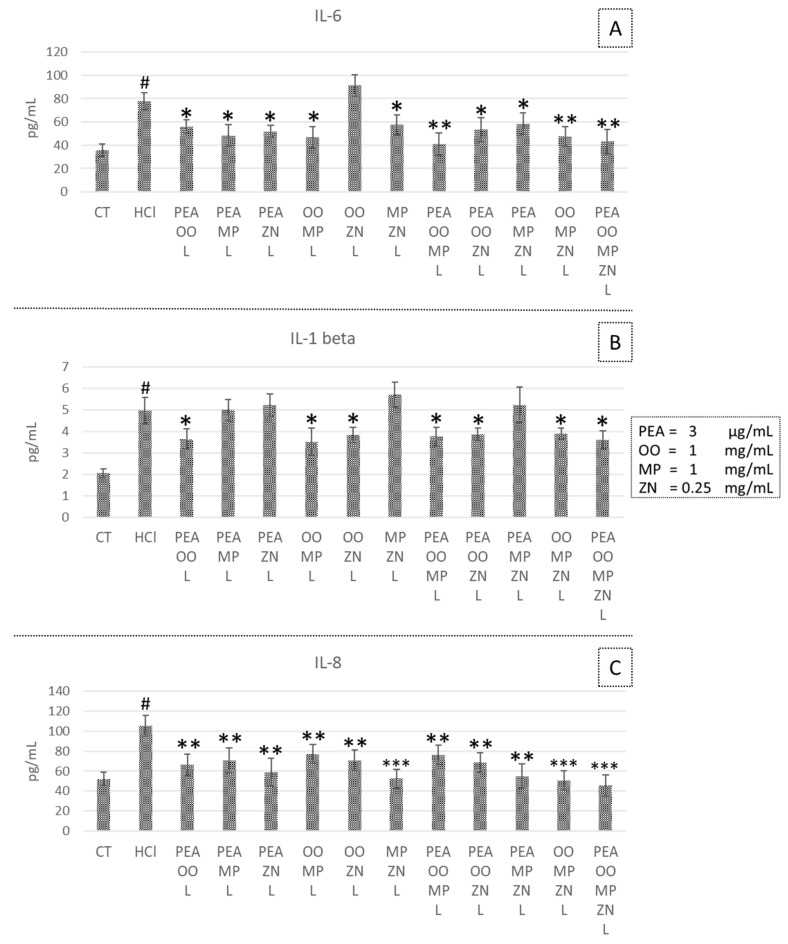
Anti-inflammatory effects of binary, ternary and quaternary mixtures at low concentrations, analyzed by measuring the secretion of the pro-inflammatory cytokines IL-6 (**A**), IL-1β (**B**) and IL-8 (**C**) after acid exposure. CT, control; HCl, acid-exposed positive control; PEA, palmitoylethanolamide, OO, *Olea europaea* and *Opuntia ficus-indica* extract; MP, *Musa × paradisiaca*; ZN, *Zingiber officinale*. Concentration values are listed in the figure legend. * *p* < 0.05, ** p < 0.01 and *** *p* < 0.001 compared to the HCl-stimulated positive control. # *p* < 0.001 compared to the negative untreated control (CT).

Binary, ternary and quaternary mixtures were able to significantly decrease IL-6 and IL-8 secretion in HCl-treated CP-B cells (Figure 4A,C). A different pattern was observed for IL-1β, which showed a statistically significant reduction only in the presence of OO (Figure 4B).

In the LPS model, no significant reduction in cytokine secretion was observed for binary, ternary and quaternary mixtures (see Supplementary Figure S2).

Based on the combination index (Ci) calculated with the software SynergyFinder^®^, in the HCl model, a synergistic effect on IL-1β modulation was detected in binary mixtures at the lower concentrations of PEA and OO (Ci = 0.82); PEA and ZN (Ci = 0.64) and in the quaternary mixture of PEA, OO, MP and ZN (Ci = 0.70). An additive effect was detected between OO and MP (Ci = 1).

A synergistic effect was also detected in the quaternary mixture of PEA, OO, MP and ZN (Ci = 0.3) for IL-8 secretion. No synergistic or additive effect between compounds was detected in IL-6 cytokine secretion.

## 4. Discussion

In this study, we used two different in vitro models, both based on the CP-B cell line. This Barrett’s esophagus cell line is negative for gastric mucin coat, and thus is particularly vulnerable to HCl damage.

The first inflammatory model was obtained by exposing esophageal cells to an HCl-containing medium which, although devoid of the protease component, constitutes a strong insult to the cells. This is a modification of the model originally proposed by Rafiee and collaborators [23] based on the exposure of different esophageal cells to HCl for 20 min. In the HCl model, inflammation is mainly mediated by an increase in NF-kB activity [23]. In our CP-B cells, after a 20 min HCl exposure, cell death increased up to 10%, and the monolayer started to show gaps. We then chose to reduce exposure time so as not to affect cell number and viability, as confirmed by a cell count and MTT test (less than 2% of cell death). This shorter exposure to HCl stimulated a mild inflammation of CP-B cells, with cytokine secretion that increased significantly but modestly.

In GERD, Gram-negative bacteria dominate the esophageal microbiome, producing considerable amounts of lipopolysaccharide (LPS) [26]; we therefore decided to use a model of inflammation based on CP-B cell exposure to this bacterial toxin. LPS has been widely used to stimulate inflammation in vitro, acting mainly by activation of the toll-like receptor 4 (TLR4) [27]. In our model, LPS was able to induce strong inflammation and to dramatically raise the secretion of all of the cytokines we measured.

The results obtained by testing our natural compounds in the two models were completely different, and these differences can be explained by both the pharmacological proprieties of the tested compounds and the differences in the molecular mechanism underlying the inflammation onset between HCl and LPS.

In the HCl model, all treatments except ZN showed an anti-inflammatory effect and were able to reduce IL-6, IL-8 and IL-1β secretion. The protective effects of OO and MP in GERD were already known [17]; in our experiments, we analyzed their direct effect on esophageal mucosa. Moreover, we detected an anti-inflammatory effect of PEA in esophageal cells. PEA has been identified as an agonist of the PPAR-α receptor, whose activation promotes NFkB inactivation, thus reducing the transcription of pro-inflammatory genes [28,29]. Moreover, PPAR-α showed a cytoprotective effect in gastric ulcers [30]. Another putative target of PEA is the GPR55 receptor [31], widely distributed in the gastrointestinal tract and considered a possible target for the treatment of inflammation. However, GPR55 pharmacology is still not fully understood and there are no specific data on its expression in the esophageal mucosa [32]. PEA is also capable of indirectly activating CB1 and CB2 receptors [28], whose implication in inflammatory pathways in the GI tract is becoming increasingly evident [33]. In esophageal mucosa, CB1 expression was described by our research group in GERD patients, and a higher expression of this receptor was detected in non-erosive patients compared to erosive ones [34]. Another study showed that the CB1 agonist delta9-tetrahydrocannabinol was able to control esophageal motility and reduce the transient LES relaxations associated with GERD onset, although this effect is likely to be mediated by the central nervous system [35].

The effect of OO and MP seems to be linked to their ROS scavenging activity [36,37,38], which protects cells and tissues from increased inflammatory damage [36].

*Olea europaea* has previously been used at a concentration of 0.5 mg/mL in experiments on monocytes stimulated with LPS [36]. In our experiments, we selected a dosage of the mixture (OO) corresponding to approximately 0.5 mg/mL of the two active ingredients for the lower dosage. On the other hand, MP has shown in vitro antioxidant properties at doses ranging between 0.5 and 1 mg/mL [38], so in our experiments, we used 1 mg/mL as the lower dose to be tested. Overall, these doses showed efficacy in the HCl-induced model of inflammation but not in the LPS model.

The pharmacological activity of PEA, OO and MP can explain the absence of a significant effect in the LPS model. In fact, TLR4 is not a direct target of PEA and, with regard to OO and MP, their effects are not directed toward specific inflammatory targets, but they are exerted indirectly by scavenging reactive oxygen species [39,40]. Our hypothesis is that, overall, the pharmacological activities of PEA, OO and MP at the tested doses were not adequate to counteract the strong inflammation induced by LPS, since their effects are not exerted directly on LPS-activated pathways. In support of this hypothesis, we observed that the only mild anti- inflammatory effect in the LPS model was obtained with ZN treatment, which is the only compound containing 6-gingerol and zerumbone that are able to interfere with pro-inflammatory cytokine synthesis induced by the activation of the arachidonate cascade due to LPS treatment [41,42]. Thus, it is not surprising that ZN decreased only IL-6 synthesis in the LPS model, which is a cytokine highly expressed by CP-B cells and, more generally, in the condition of GERD [43]. On the other hand, HCl exposure does not seem to mainly activate the arachidonate cascade, but rather induces transcriptional regulation of IL-6 and IL-8 expression involving MAPK and PKC signaling, and NF-κB activation [23]. This could explain why ZN extract had no effect on the inflammation induced by the HCl exposure.

Analyses of the anti-inflammatory effects of binary, ternary and quaternary mixtures of the tested compounds were performed to evaluate their combined effects. Results showed an efficacy of the combined compounds in the HCl model, but not in the LPS ones, confirming that these compounds are able to counteract acidification damages typical of GERD, but not stronger inflammatory stimuli.

In the HCl model, we detected synergistic effects on IL-1β modulation in binary mixtures between PEA and OO or ZN. This synergistic action on IL-1b appears to be particularly interesting because in GERD, the expression of this cytokine appears to be selectively increased both in erosive esophagitis (ERD) and in non-erosive esophagitis (NERD) [43]. Also, the quaternary mixture of PEA, OO, MP and ZN showed synergistic effects on IL-1β modulation. This quaternary mixture also shows synergistic activity towards the synthesis of IL-8, which, however, appears to be a cytokine differentially expressed in Barrett’s esophagus and less involved in the pathogenic mechanisms of ERD and NERD.

The single activities of PEA, ZN, MP, *Opuntia ficus-indica* and *Olea europaea* on different inflammatory settings have been already studied both in vitro and in vivo. Moreover, the beneficial effects of OO have been studied in patients with GERD, leading to good clinical results [18,19]. However, there was a lack of studies verifying their additive or synergistic effects.

For PEA, direct and indirect molecular targets, pharmacokinetics, and neuroprotective, anti-inflammatory and analgesic effects have been recognized [28]. Regarding the gastrointestinal tract, its analgesic effect has been studied in patients with IBS [44].

The bioactive compounds of ZN were also much studied and the mechanisms of its antioxidant, antinausea, anti-obesity, anticancer, anti-inflammatory action are known [45]. ZN has been also studied in patients with functional dyspepsia [45,46].

MP has shown antioxidant and antiulcer effects [47], and it was also able to induce apoptosis in colorectal cancer HT29 cells [48].

*Opuntia ficus-indica* showed anti-inflammatory and anti-ulcerative effects [49]. *Olea europaea* has antioxidant, anti-inflammatory, immunomodulatory, analgesic and gastroprotective effects, and it has demonstrated wound healing activities [50].

To our knowledge, this is the first study in which multiple natural extracts were studied in vitro, both alone and in combination, on a cell line derived from human Barrett’s esophageal metaplasia, and their inflammatory profiles were evaluated. The novelty of this study is to suggest the use of PEA, alone or combined to other natural extracts, to treat GERD. We are aware that our study has some limitations. First, the experimental scheme considers only in vitro studies on a single cell line. Furthermore, the results observed were obtained from only two types of experimental data obtained from the MTT assay and from the cytokine measurements, although they were all conducted in quadruplicate.

## 5. Conclusions

Overall, our results provide the rationale for the use of PEA, alone or even better in combination with other natural compounds such as ZN, OO and MP, in the long-term management of GERD in all its endoscopic phenotypes (erosive, non-erosive and Barrett’s esophagus). Future experiments could be conducted in vivo on GERD animal models to confirm our in vitro data. Nevertheless, GERD models in mice have several limitations. A clinical trial on GERD patients, using PEA and plant extracts could be carried out to confirm the efficacy of PEA possibly in association with the other plant extracts we tested. On the other hand, animal models could help to define the dosages at which these actives could be effective in vivo.

## Figures and Tables

**Table 1:** Binary, ternary and quaternary mixtures. PEA, Palmitoylethanolamide, OO, *Olea europaea* and *Opuntia ficus-indica* extract; MP, *Musa × paradisiaca*; ZN, *Zingiber officinale*.

Binary mixtures	PEA 3 µg/mL+ OO 1 mg/mL
	PEA 3 µg/mL + MP 1 mg/mL
	PEA 3 µg/mL + ZN 0.25 mg/mL
	OO 1 mg/mL + MP 1 mg/mL
	OO 1 mg/mL + ZN 0.25 mg/mL
	MP 1 mg/mL+ ZN 0.25 mg/mL
Ternary mixtures	PEA 3 µg/mL + OO 1 mg/mL + MP 1 mg/mL
	PEA 3 µg/mL+ OO 1 mg/mL + ZN 0.25 mg/mL
	PEA 3 µg/mL+ MP 1 mg/mL + ZN 0.25 mg/mL
	OO 1 mg/mL + MP 1 mg/mL+ ZN 0.25 mg/mL
Quaternary mixture	PEA 3 µg/mL+ OO 1 mg/mL + MP 1 mg/mL + ZN 0.25 mg/mL

## Data Availability

The original contributions presented in the study are included in the article/Supplementary Materials, further inquiries can be directed to the corresponding author.

## Supplementary Materials

The following supporting information can be downloaded at: https://www.mdpi.com/article/10.3390/life14101221/s1, Figure S1: Morphology of cultured CP-B cells; Figure S2: Anti-inflammatory effects of mixtures evaluated by using the LPS model.
