# Supplementary material for: Palmitoylethanolamide (PEA) for Prevention of Gastroesophageal Inflammation: Insights from In Vitro Models
Source: Life (Basel). 2024 Sep 24;14(10):1221. doi: 10.3390/life14101221 (PMC11508466; doi:10.3390/life14101221)
Supplement: Supplementary file 1 [file life-14-01221-s001.zip › life-3134178-supplementary.pdf]

life

MDPI

Supplementary figures 1

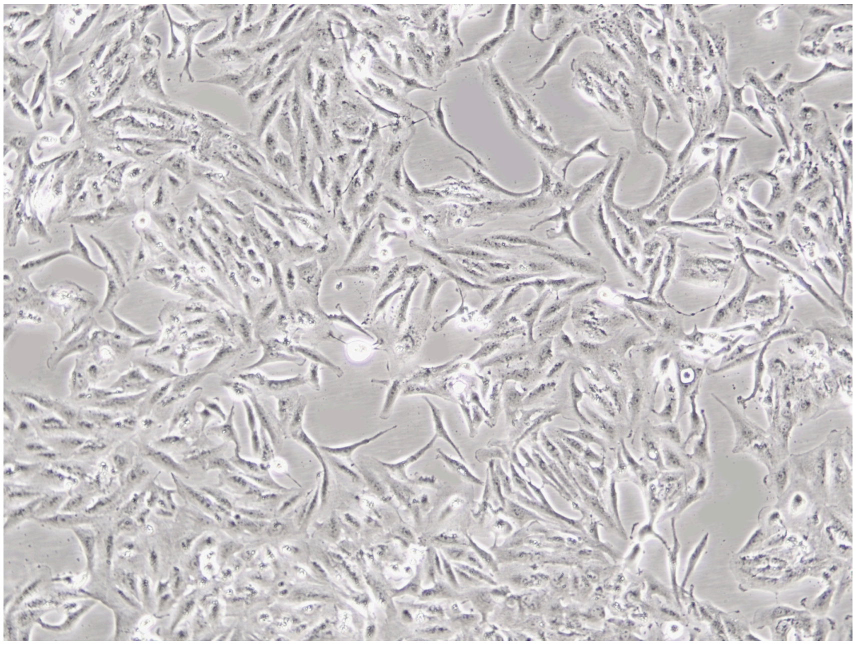

Figure S1. Morphology of cultured CP-B cells at 70% of confluence, acquired at total magnification 100X, with an inverted phase-contrast microscope Optika IM-3.

Supplementary figures 2

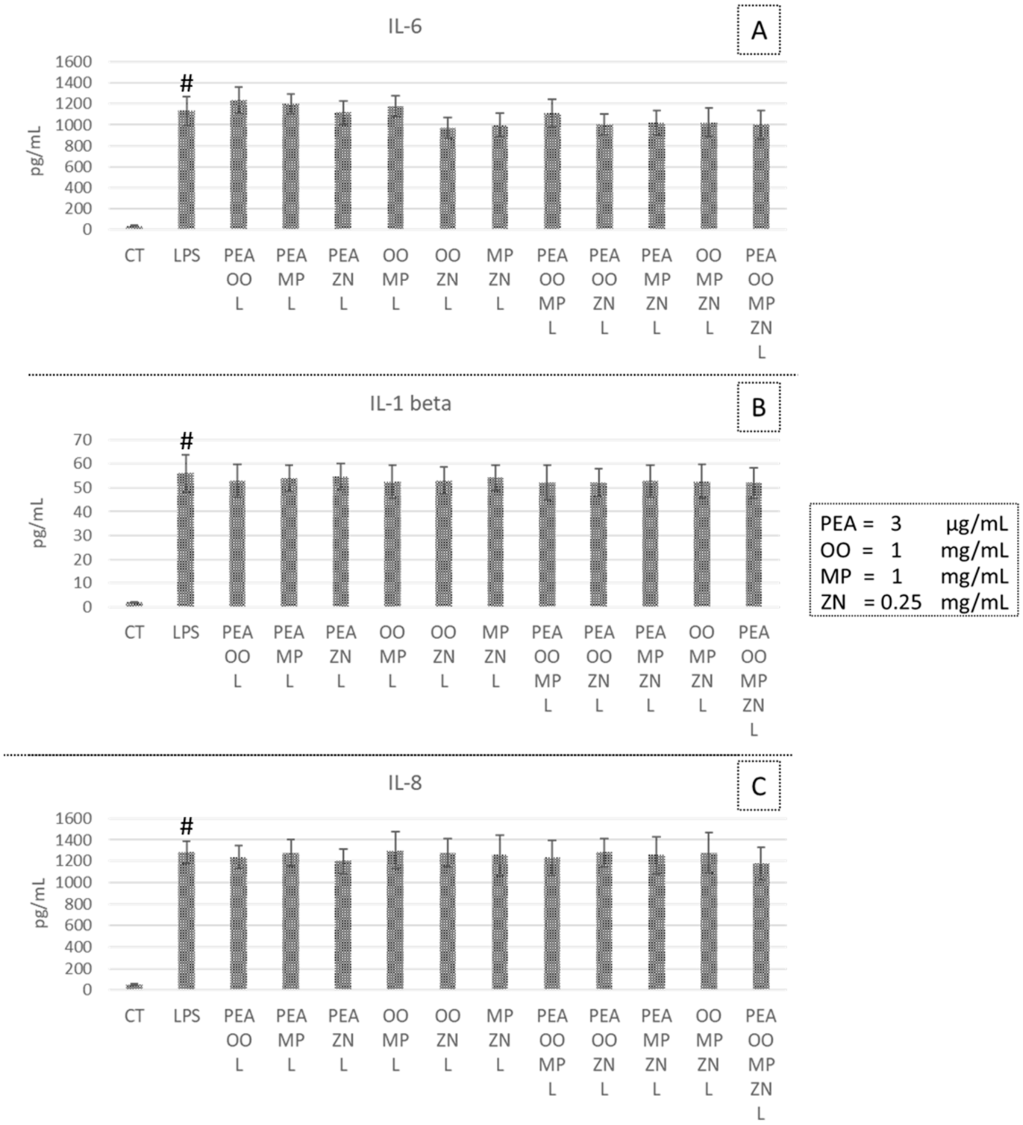

Figure S2. Anti-inflammatory effects of binary, ternary and quaternary mixtures at low concentrations, analysed by measuring the secretion of the pro-inflammatory cytokine IL-6 (panel A), IL-1β (panel B) and IL-8 (panel C) after LPS stimulation. CT, control; LPS, Lipopolysaccharide positive control; PEA, Pamitoylethanolamide, OO, *Olea europaea* and *Opuntia ficus-indica* extract; MP, *Musa x paradisiaca*; ZN, *Zingiber officinale*. Concentration values are listed in the figure legend. #=$p<0.001$ compared to the negative untreated control (CT).
